# *madd-4* plays a critical role in light against *Bursaphelenchus xylophilus*

**DOI:** 10.1038/s41598-022-19263-9

**Published:** 2022-08-30

**Authors:** Lifeng Zhou, Bicheng Sheng, Tianyuan Zhang, Wenyi Liu, Kai Guo, Hongshi Yu, Liqun Bai, Jiafu Hu

**Affiliations:** 1 College of Forestry and Biotechnology, Zhejiang A & F University, Hangzhou, 311300 China; 2 School of BioSciences, The University of Melbourne, Parkville, VIC 3010 Australia

**Keywords:** Molecular biology, RNAi

## Abstract

*Bursaphelenchus xylophilus* is a notorious invasive species, causing extensive losses to pine ecosystems globally. Previous studies had shown that the development of *B. xylophilus* was seriously suppressed by light. However, the mechanism involved in the inhibition is unknown. Here, it is the first report that *Bxy-madd-4* is a light-regulated gene, plays a potential role in *B. xylophilus* in responding to the blue light. Transcriptome sequencing revealed that the expression level of *Bxy-madd-4* declined by 86.39% under blue light. The reverse transcription quantitative real-time PCR results were in accord with the transcriptome sequencing, confirming the expression level of *Bxy-madd-4* was suppressed by blue light. *Bxy-madd-4* promoter::mCherry reporter constructed in *Caenorhabditis elegans* were utilized to mimic the spatiotemporal expression patterns of *Bxy-madd-4*. *Bxy-madd-4A* promoter activity had a strong continuity throughout all development stages in *C. elegans*. Further RNA interference indicated that only 36.8% of the *Bxy-madd-4* dsRNA treated embryos were hatched. Moreover, 71.6% of the hatched nematodes were abnormal, such as particles on the body surface and concave tissues. Our findings contribute towards a better understanding of the mechanism of light against the destructive invasive nematode, providing a promising hint for control of the destructive invasive nematode.

## Introduction

*Bursaphelenchus xylophilus* is the causal agent of the pine wilt disease causing extensive economic and ecological losses globally^1,2^. It was first found in Japan since the beginning of the twentieth century and spread into the neighboring countries in the late 1980s^3^. After devastating millions of pine trees in Asian countries, the nematode spread into Europe in the late 1990s^4^ and caused worldwide concern. The infection cycle of the pinewood nematode is exceptionally complicated. It feeds on parenchyma cells and propagates in the resin canals of the living pine trees^5^. At 25 °C, the embryonic development of the nematode requires ~ 20 h (h). The second-stage juvenile (J2) emerge (the J1 within the egg) after embryogenesis and begin their feeding activity^6^. In the postembryonic development, J2 undergoes three additional molts before becoming adult, which requires ~ 4 days (d) at 25 °C^7^. Within a few months, the host pine tree will wilt and die due to infection by the nematode^8^. The quick wilt and death of the host could be caused by the collapsing of the water conduction tissues caused by the fast losing of the parenchyma cells^9^. After the host dies, the nematode enters a fungivorous stage, feeding on the fungi that invaded the wilting pine tree^5^. *B. xylophilus* could not deviate from the host pine trees independently and, but depend on the insect vectors (*Monochamus* spp.)^10^. *B. xylophilus* intrudes into the insect vectors when the *Monochamus* beetles pupate in the pupal chambers and invade the new host pine trees when the adult beetle feeds or oviposits on pine trees^11^. Thus, at least four organisms are involved in the infection cycle of this disease: host tree, pine wood nematode as the fatal pathogen, insect vector, and fungi growing in the withering tree. To this date, although a large number of studies have been carried out to controlling pine wood nematode, no approved countermeasures have been developed. In fact, it is still rampant, causing huge economic losses and ecological threats in the past several years worldwide.

An early study in Japan described that pine wilt disease development was significantly suppressed under white light illumination^12^. Recently, we found that population growth of *B. xylophilus* was significantly suppressed by the continuous illumination of white light. In general, the light sensation is vital for all organisms, from microorganisms to humans^13^. Besides the light irritation that makes changes in the metabolism and avoids the responses in microorganisms, it stimulates attraction responses in plants and induces non-visual or visual perception in animals^14^. Six photoreceptor families including cryptochromes, phototropins, BLUF proteins, rhodopsins, phytochromes, and xanthopsins, have been widely acknowledged to mediate light signal transduction in nature^15^. The first three have different flavin-based photochemistry, whereas the last three belong to isomerization of retinal, p-coumaric acid, and phytochromobilin. However, very few studies have examined the effects of light on nematodes. The possible molecular mechanisms of light-suppressed growth and development are not clear in nematodes.

In order to clarify the molecular basis of the light suppression, we conducted a comparative transcriptome analysis in *B. xylophilus* with light and dark treatment, using the RNA-Seq technology^16^. The results showed that the expression level of muscle arm development defective gene (*madd*) was strongly suppressed by the blue light. MADD-4 is orthologous to a mammalian gene named ADAMTSL1 (a disintegrin-like and metalloprotease domain with thrombospondin type I motifs-like)^17^. ADAMTSL proteins are homologous with ADAMTS (a disintegrin and metalloprotease with thrombospondin domains) proteins but out of metalloprotease and disintegrin amino-terminal domains^18^. ADAMTS is a family of extracellular proteases found in mammals and invertebrates, playing crucial roles during embryonic development and angiogenesis^19^. *madd-4* was firstly identified from *Caenorhabditis elegans* by a forward genetic screen for muscle arm extension mutants^20^. In *C. elegans*, the protein of *madd-4*, MADD-4, is secreted by the ventral and dorsal nerve cords to induce extending sensory axons and muscle arms^17^. The activity of MADD-4 is dependent on the netrin receptor, UNC-40, which functions cell-independently to guide membrane expansion^17^. Furthermore, MADD-4 and neurexin redundantly control the gathering of postsynaptic GABA receptors^21^.

In the current study, we found that blue light inhibits the early embryo development of *B. xylophilus*. The expression level of *Bxy-madd-4* showing from both RT-qPCR and RNA-seq was dramatically dropped after the irritation of the blue light during embryogenesis. The lethal and retardation phenotype of embryos were found after knockdown of the expression level of *Bxy-madd-4* by RNA interference (RNAi). Therefore, our data supports the fact that that *Bxy-madd-4* plays a critical role in maintaining normal embryogenesis to defend blue light in *B. xylophilus*. The knowledge resulting from *Bxy-madd-4* would provide a hopeful clue to restraining the population growth to therefore control the invasive nematode.

## Results

### Effects of different light sources on the development of *B. xylophilus*

**Figure 1:**
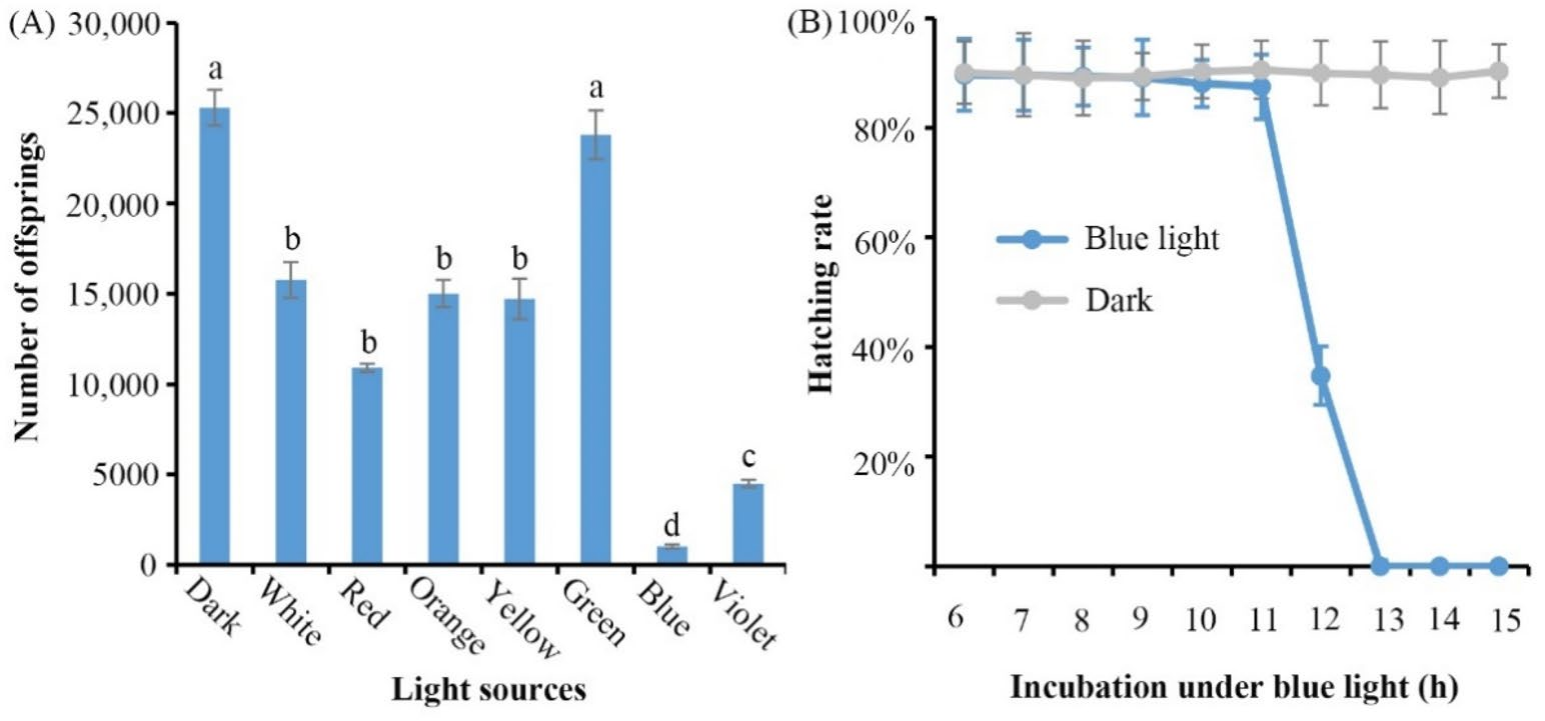
Effects of lights on the development of *B. xylophilus*. (**A**) Ten pairs (10♀ + 10♂) of virgin adult males and females were inoculated onto the mycelia of *Botrytis cinerea* and cultured under different light-emitting diode (1000 lx) at 25 °C for 7 days. The eggs incubated in dark conditions were used as a control. Different lower case letters above columns indicate statistical differences at P < 0.05. (**B**) Synchronized eggs were incubated under blue light-emitting diode (1000 lx) and dark conditions at 25 °C, for 7, 10, and 13 h, respectively. Each experiment was conducted with nine replicates. Error bars represent the standard error of the mean.

Continuous illumination with white light has been demonstrated to effectively suppress the population growth of *B. xylophilus*^12^. Therefore, we undertook a series of experiments to assess the ability of different homogeneous light to disrupt development of *B. xylophilus*. Figure 1A shows that the population growth of *B. xylophilus* was significantly inhibited under different homogeneous light-emitting diode (1000 lx). The offspring were significantly reduced by 37–96% within 7 days of treatment with the different homogeneous lights. There was a > 96% reduction in the number of offspring treated with blue light. To ensure that the reduction of offspring was due to blue light, synchronized eggs were placed under blue light for different time periods. As shown in Fig. 1B, the hatching rates were sharply declined when the time reached under the blue light up to 11 h; no eggs could hatch once they had been under the blue light for over 13 h. The results clearly demonstrate that the effectiveness of blue light is able to suppress the population growth of *B. xylophilus*.

### Sequence analysis of *Bxy-madd-4*

**Figure 2:**
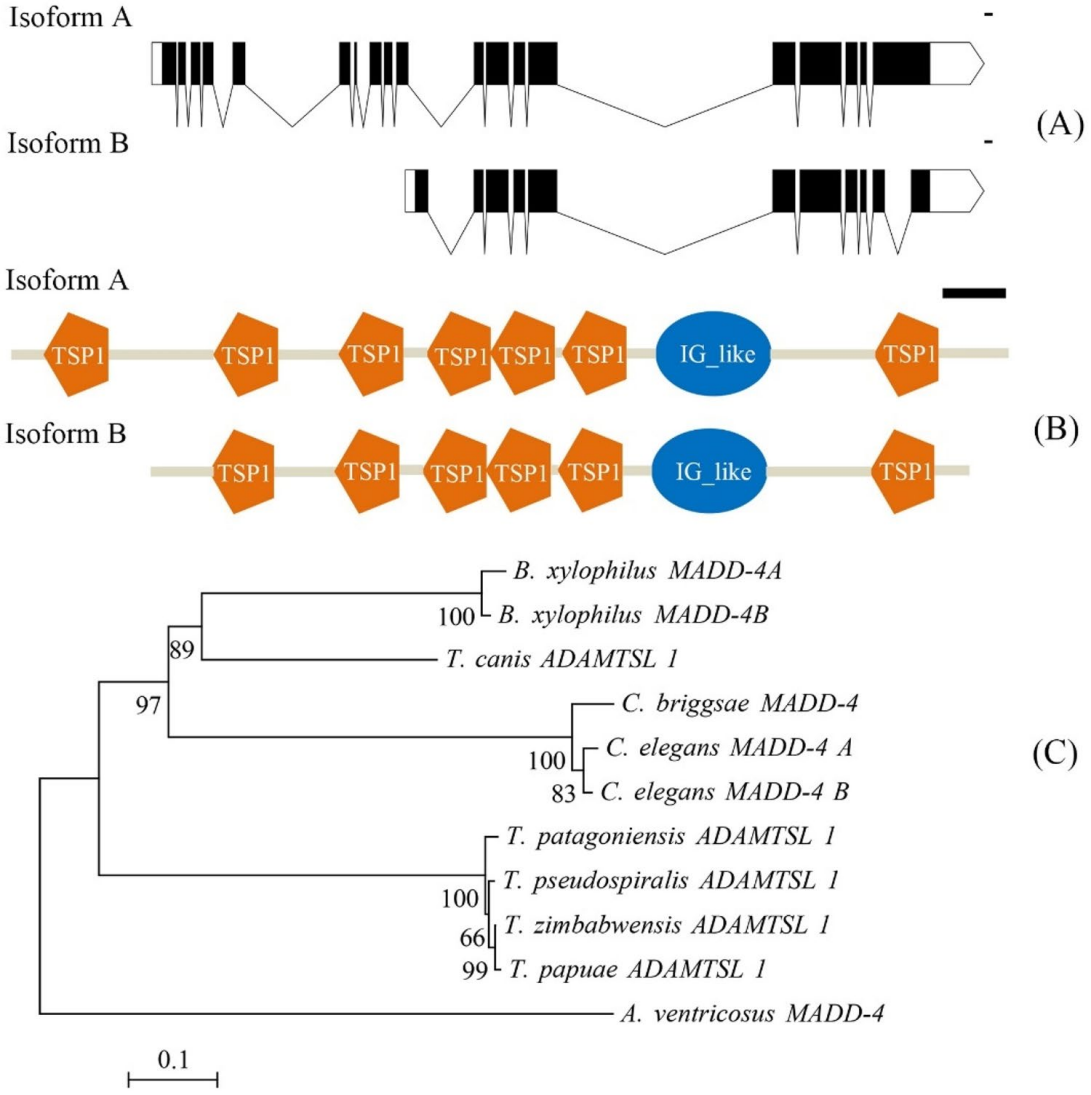
Sequence characteristics of *Bxy-madd-4*. (**A**) The structures of *Bxy-madd-4*. Exons are indicated as boxes while introns are indicated as a line; white boxes indicate untranslated regions; black boxes indicate coding sequences; scale bar, 100 bp. (**B**) Relative locations of sequence motifs with putative functions are marked. TSP1, thrombospondin type-1; immunoglobulin-like, IG-like domain; scale bar, 100 aa. (**C**) Phylogenetic analysis of ADAMTSL1 from different nematode species. Clusters were evaluated using a nonparametric bootstrap with one thousand replications. Scale bar, 0.1 substitutions per position. ADAMTSL 1: *Toxocara canis*, KHN81567.1; *Trichinella patagoniensis*, KRY18462.1; *Trichinella pseudospiralis*, KRX95259.1; *Trichinella zimbabwensis*, KRZ14515.1; *Trichinella papuae*, KRZ70098.1. MADD-4: *Caenorhabditis briggsae*, CAP31380.2; *Araneus ventricosus*, GBM34214.1. MADD-4A: *Caenorhabditis elegans*, NP_492405.3; *Bursaphelenchus xylophilus*, MT769319. MADD-4B: *C. elegans*, NP_871884.1; *B. xylophilus*, MT769320.

*Bxy-madd-4* had two isoforms, *Bxy-madd-4A* (GenBank accession number MT769319*)* and *Bxy-madd-4B* (GenBank accession number MT769320) (Fig. 2A). Isoform-A contained 75 bp of 5′ untranslated regions (UTRs), 340 bp of 3′ UTRs, and had an open reading form (ORF) of 3375 bp, encoding a protein of 1125 amino acids. Isoform-B contained 88 bp of the 5′ UTRs and 161 bp of the 3′ UTRs and had an ORF of 2169 bp, encoding 723 amino acids. The putative functional motifs of isoform-A included six N-terminal TSP1 (thrombin sensitive protein, thrombospondin-1) motif repeats, an IG-like (immunoglobulin-like) domain, and a C-terminal TSP1 motif. Isoform-B was similar to isoform-A, except that it lacked an N-terminal TSP1 (Fig. 2B). No transmembrane helices were predicted in Bxy-MADD-4 proteins. However, it had a signal peptide, Bxy-MADD-4A signal peptide cleavage site at Ala_18_-Ile_19_, whereas Bxy-MADD-4B signal peptide cleavage site at Ala_17_-Val_18_. The phylogenetic tree of ADAMTSL1 protein showed that Bxy-MADD-4 had the highest homology level with ADAMTSL1 of the *Toxocara canis*, and assembled into a valid cluster with those of other nematodes (Fig. 2C). The molecular formula of Bxy-MADD-4A was estimated to be C_5363_H_8471_N_1613_O_1662_S_89_ with a molecular weight of 124.99 kDa and a predicted theoretical pI of 8.62. In contrast, the molecular formula of Bxy-MADD-4B was estimated to be C_3459_H_5487_N_1025_O_1067_S_53_ with a molecular weight of 80.20 kDa and a predicted theoretical pI of 8.58. The instability index (II) of Bxy-MADD-4A and Bxy-MADD-4B was computed to be 48.24 and 44.85, indicating that they might be unstable proteins.

### Validation of gene expression data using RT-qPCR

**Figure 3:**
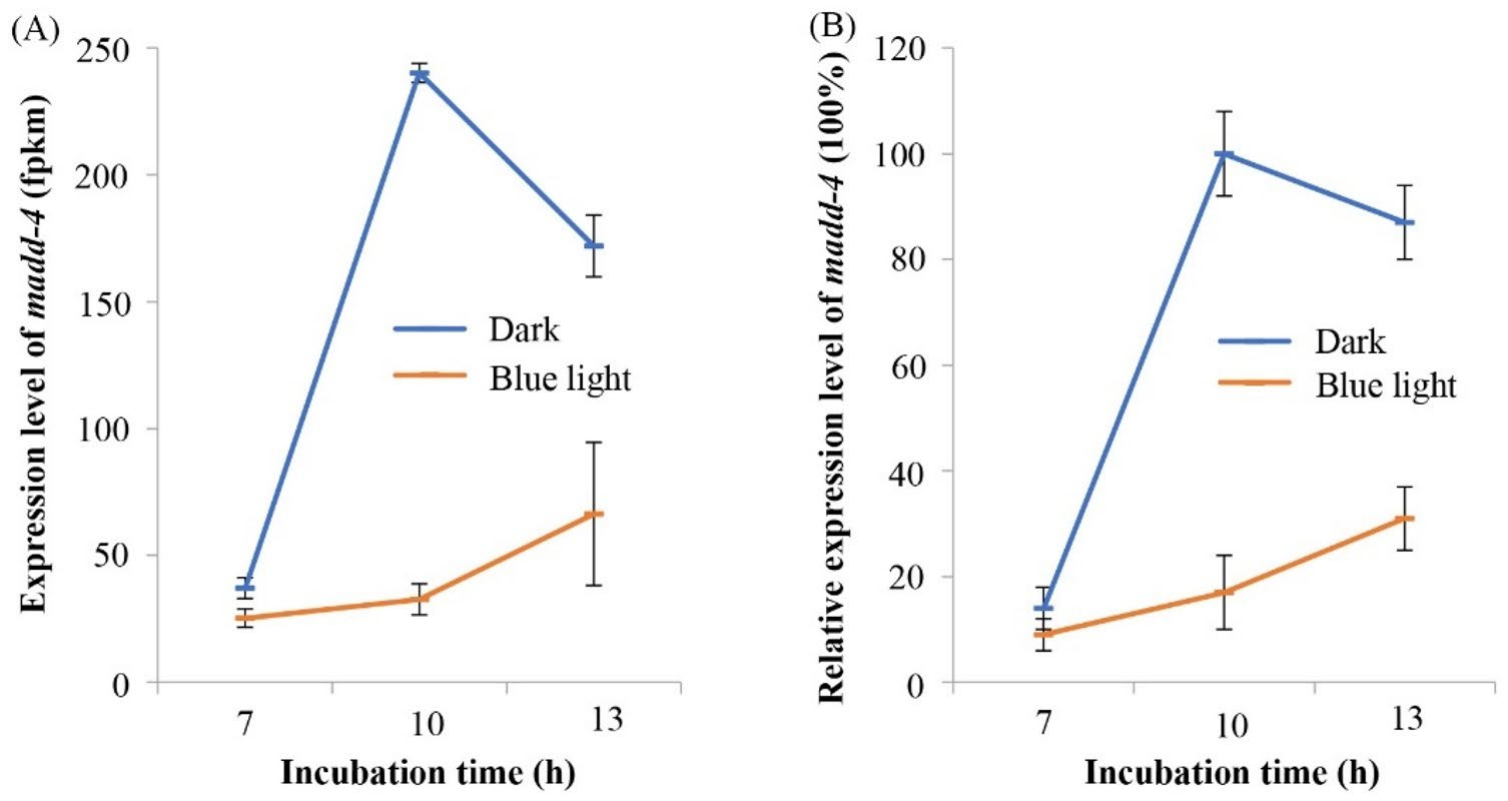
The expression levels of *Bxy-madd-4* during embryogenesis in *B. xylophilus*. Synchronized eggs were incubated under blue light-emitting diode (462 ± 6 nm; 1200 lx) and dark conditions at 25 °C, for 7, 10, and 13 h, respectively. (**A**) All FPKM values of *Bxy-madd-4* obtaining from RNA-Seq results. Each sample has three biological replicates. (**B**) The relative expression level of *Bxy-madd-4* was determined by RT-qPCR, normalized with *β-actin* and *tbb-2* as the reference genes. The expression level of 10 h in dark conditions was appointed as 100%. RT-qPCR reactions were performed in triplicate. Error bars represent the standard error of the mean.

RNA-seq and RT-qPCR were used to examine the expression level of *Bxy-madd-4* during embryogenesis in *B. xylophilus* under blue light and dark conditions. The FPKM (fragments per kilobase of exon model per million mapped fragments) data (Supplementary Table S1) of *Bxy-madd-4* in our previous RNA-seq results showed a higher expression in 10 h than that of 7 h and 13 h in dark conditions, and the blue light effectively decreased the expression level (Fig. 3A). Compared to the dark conditions, the expression level of *Bxy-madd-4* under blue light was declined by 31.83% in 7 h, 86.39% in 10 h, and 61.41% in 13 h. The RT-qPCR results were consistent with the expression patterns obtained from RNA-Seq results (r = 0.983 for dark conditions and r = 0.981 for blue light conditions), confirming that the expression level of *Bxy-madd-4* was suppressed by blue light (Fig. 3B).

### Spatial and temporal characteristics of *Bxy-madd-4*

**Figure 4:**
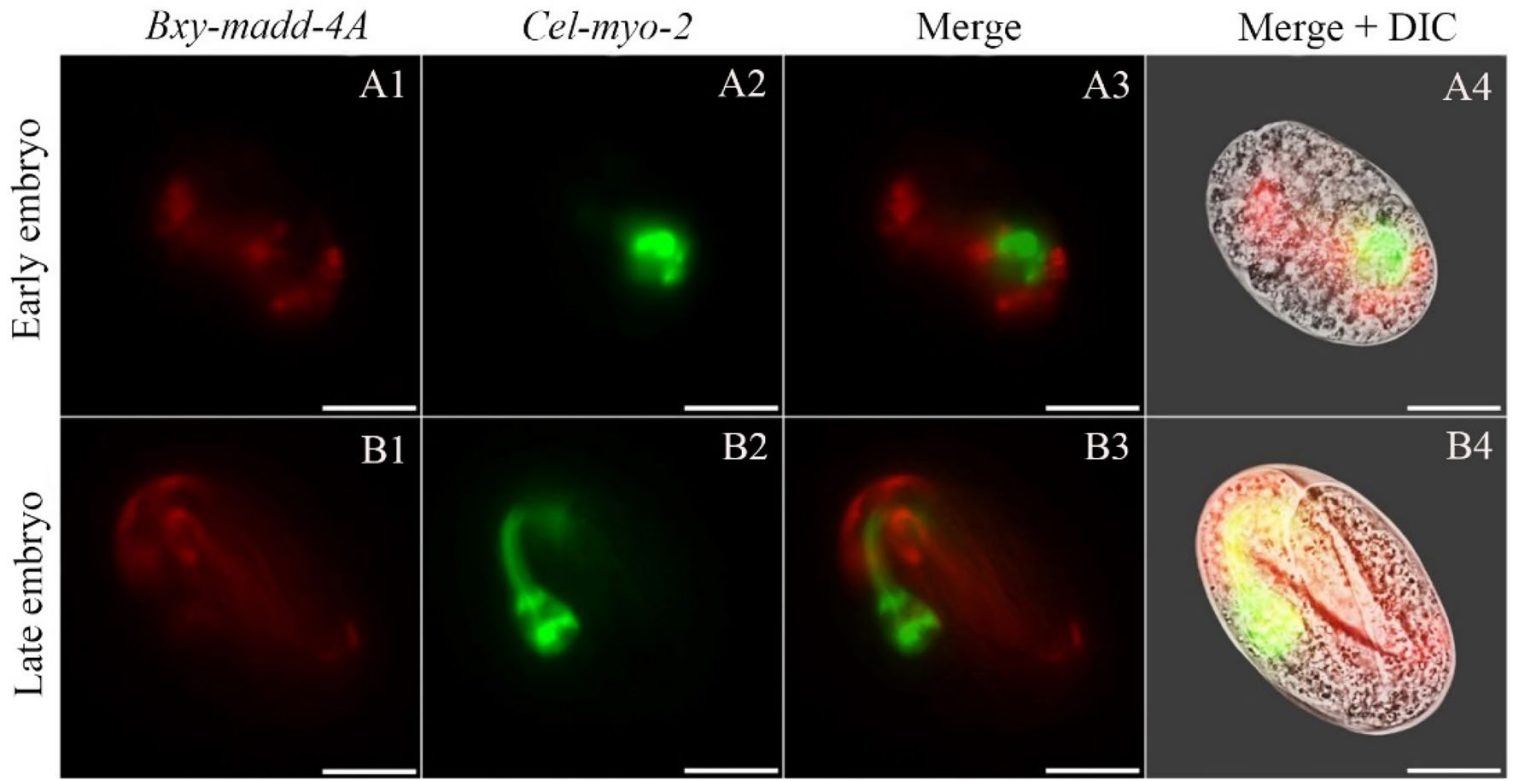
Localization of *Bxy-madd-4A* on translational level via transgenic *C. elegans* during embryonic development. The expression patterns of mCherry (red) under control of *B. xylophylus madd-4A* promoter and green fluorescent protein gene (green) was driven by *C. elegans* promoter *myo-2* as a marker for pharynx development. Fluorescent (A1–A3 and B1–B3) and differential interference contrast (DIC) merged (A4 and B4) microscope images of the second filial generation transgenic *C. elegans* embryos carrying both *Bxy-madd-4Ap*::mCherry and *Pmyo-2*::GFP transgenes. Early embryo, raised at 20 °C for 6 h; late embryo, raised at 20 °C for12 h. In all cases, images are representative of 50 embryos. The scale bar stands for 10 μm.

To mimic the spatial and temporal expression profiles of *Bxy-madd-4* at the translational level, *Bxy-madd-4Ap* and *Bxy-madd-4Bp* were cloned into the expression vectors and injected into *C. elegans*, respectively. During early embryogenesis (6 h), *Bxy-madd-4Ap* activity was detectable in several specific areas (red) of the *C. elegans* embryo, unlike *gfp* (green), which focused on only one area (Fig. 4A1–A4). During late embryogenesis (12 h), *Bxy-madd-4Ap* activity was detectable in most parts (red) of the embryo, and the pharynx outline formed (green) (Fig. 4B1–B4).

**Figure 5:**
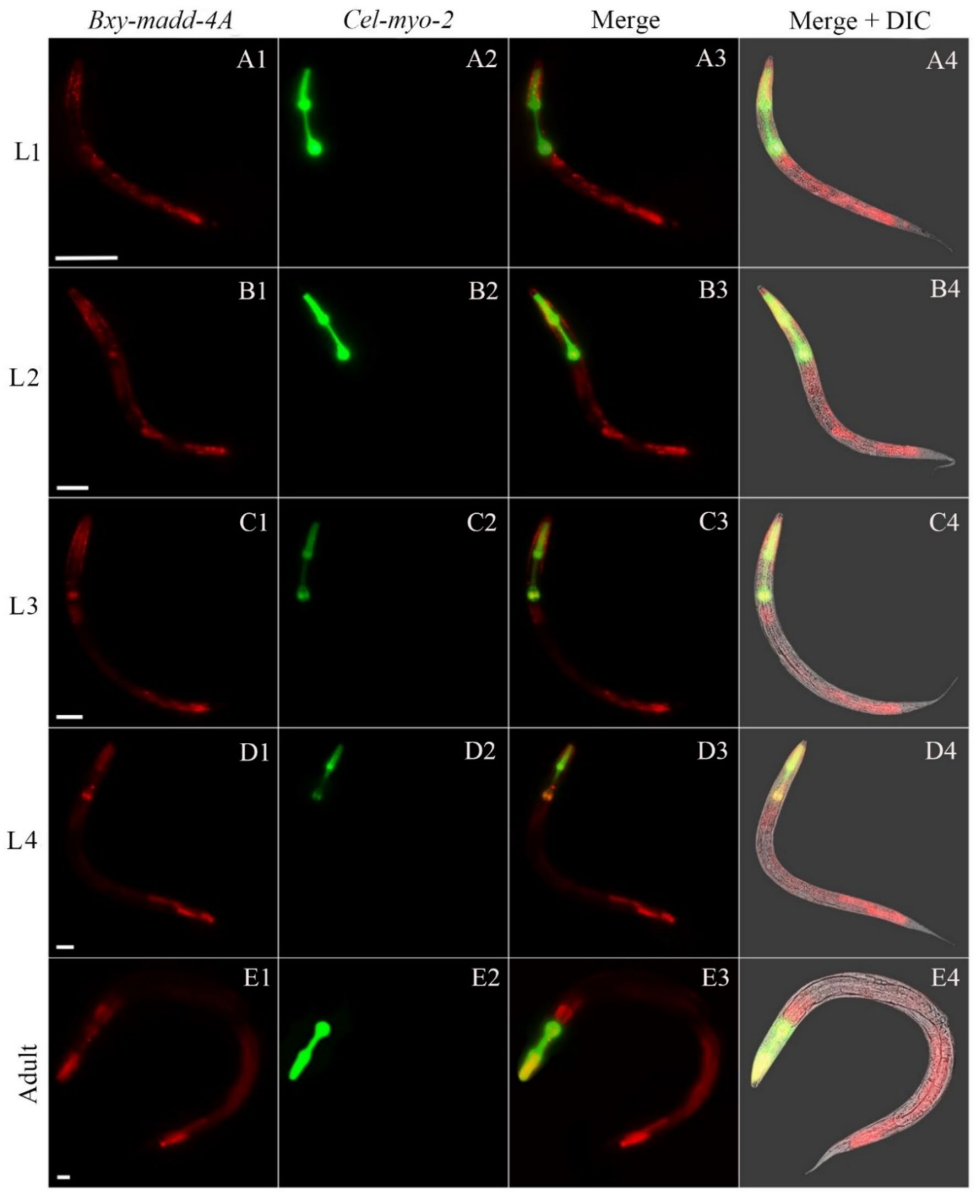
Localization of *Bxy-madd-4A* on translational level via transgenic *C. elegans* during postembryonic development. The expression patterns of mCherry (red) under control of *B. xylophilus madd-4A* promoter and green fluorescent protein gene (green) was driven by *C. elegans* promoter *myo-2* as a marker for pharynx development. Fluorescent and differential interference contrast (DIC)merged microscope images of the second filial generation transgenic *C. elegans* carrying both *Bxy-madd-4Ap*::mCherry and *Pmyo-2*::GFP transgenes. L1, first-stage juvenile (A1–A4); L2, second-stage juvenile (B1–B4); L3, third-stage juvenile (C1–C4); L4, fourth-stage juvenile (D1–D4); and adult hermaphrodite (E1–E4). In each age group, 50 nematodes were examined. The scale bar stands for 20 μm.

*Bxy-madd-4Ap* activity had a strong continuity throughout all postembryonic stages in *C. elegans*. As a marker gene for pharynx, *gfp* (green) showed a complete structure of the pharynx in the postembryonic stages (Fig. 5). In the stage of L1, the *Bxy-madd-4Ap* activity was nearly present throughout the entire body from head to tail but was weakly distributed in the pharynx areas (Fig. 5A1–A4). The *Bxy-madd-4Ap* activity in L2 was basically the same as in L1 (Fig. 5B1–B4). In L3, L4 and adult stage, the *Bxy-madd-4Ap* activity presented a somewhat different result from L1 and L2 in the middle of the worms, however it was similar in other parts (Fig. 5).

**Figure 6:**
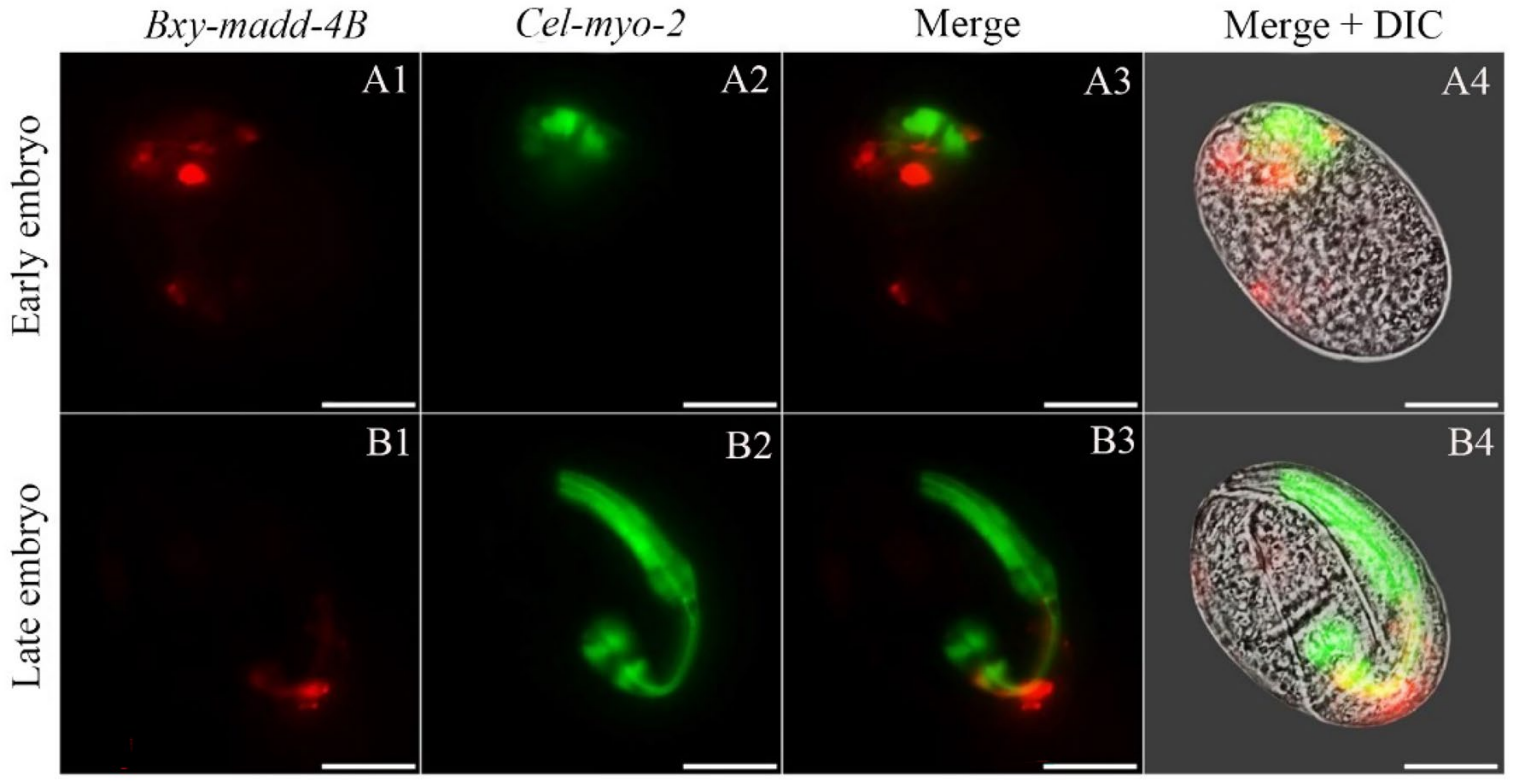
Localization of *Bxy-madd-4B* on translational level via transgenic *C. elegan*s during embryonic development. The expression patterns of mCherry (red) under control of *B. xylophylus madd-4B* promoter and green fluorescent protein gene (green) was driven by *C. elegans* promoter *myo-2* as a marker for pharynx development. Fluorescent (A1–A3 and B1–B3) and differential interference contrast (DIC) merged (A4 and B4) microscope images of the second filial generation transgenic *C. elegans* embryos carrying both *Bxy-madd-4Bp*::mCherry and *Pmyo-2*::GFP transgenes. Early embryo was raised at 20 °C for 6 h and late embryo was raised at 20 °C for 12 h. In all cases, images are representative of 50 embryos. The scale bar stands for 10 μm.

**Figure 7:**
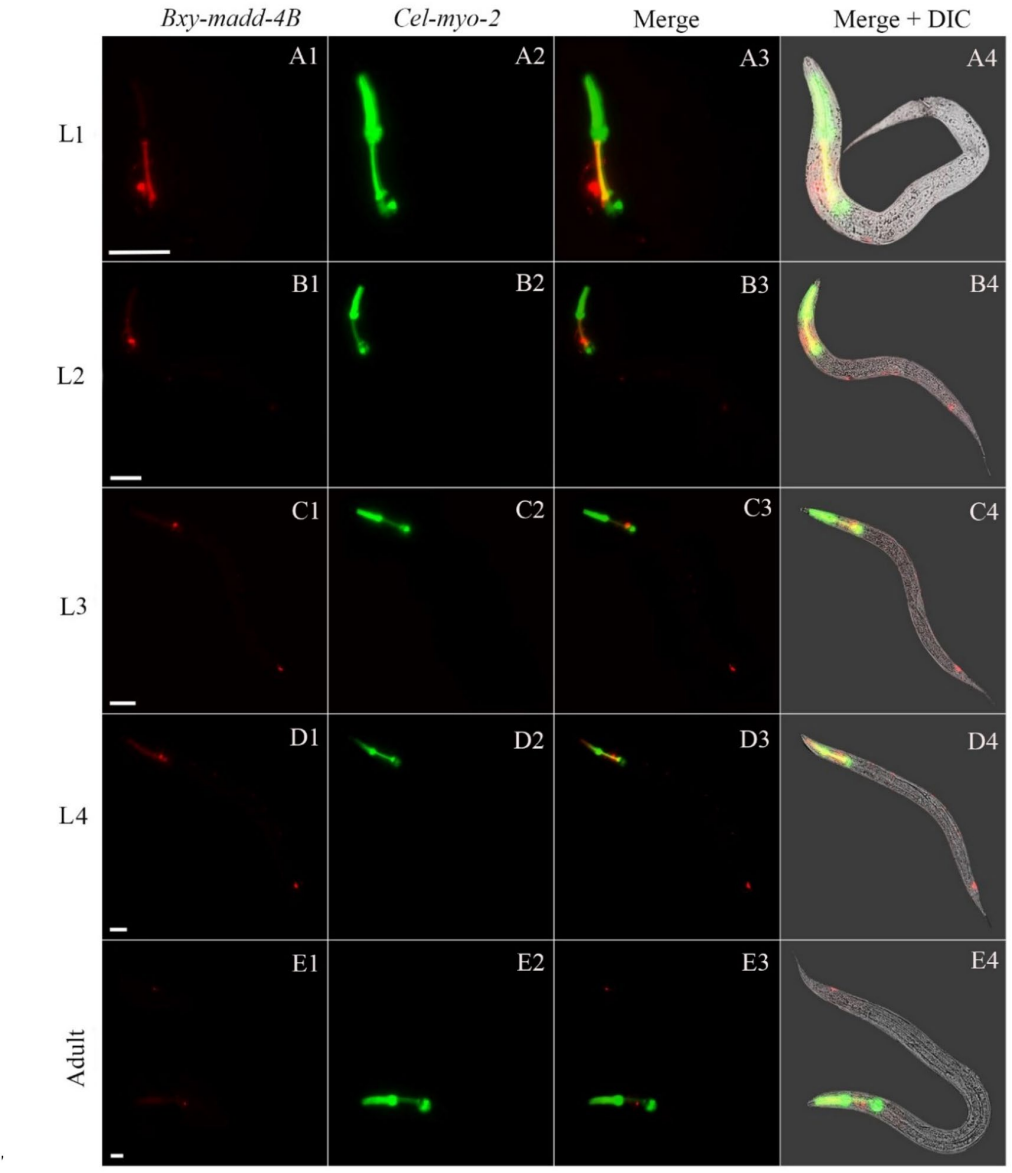
The localization of *Bxy-madd-4B* on translational level transgenic *C. elegans* during postembryonic development. The expression patterns of mCherry (red) under control of *B. xylophilus madd-4B* promoter and green fluorescent protein gene (green) was driven by *C. elegans* promoter *myo-2* as a marker for pharynx development. Fluorescent and differential interference contrast (DIC) merged microscope images of the second filial generation transgenic *C. elegans* carrying both *Bxy-madd-4Bp*::mCherry and *Pmyo-2*::GFP transgenes. L1, first-stage juvenile (A1–A4); L2, second-stage juvenile (B1–B4); L3, third-stage juvenile (C1–C4); L4, fourth-stage juvenile (D1–D4); and adult hermaphrodite (E1–E4). In each age group, 50 nematodes were examined. The scale bar stands for 20 μm.

*Bxy-madd-4Bp* activity had obvious differences with that of *Bxy-madd-4Ap* over the course of ontogeny in *C. elegans*. During the embryogenesis, *Bxy-madd-4Bp* activity was detectable in only a few areas (red) near the pharynx (green) in the *C. elegans* embryo (Fig. 6). Moreover, *Bxy-madd-4Bp* activity continued to decrease during the postembryonic development stages of *C. elegans* (Fig. 7). In the adult hermaphrodite, *Bxy-madd-4Bp* activity was only detectable as two tiny dots, one close to the pharynx and the other in the tail.

### Characterization and analysis of RNAi

**Figure 8:**
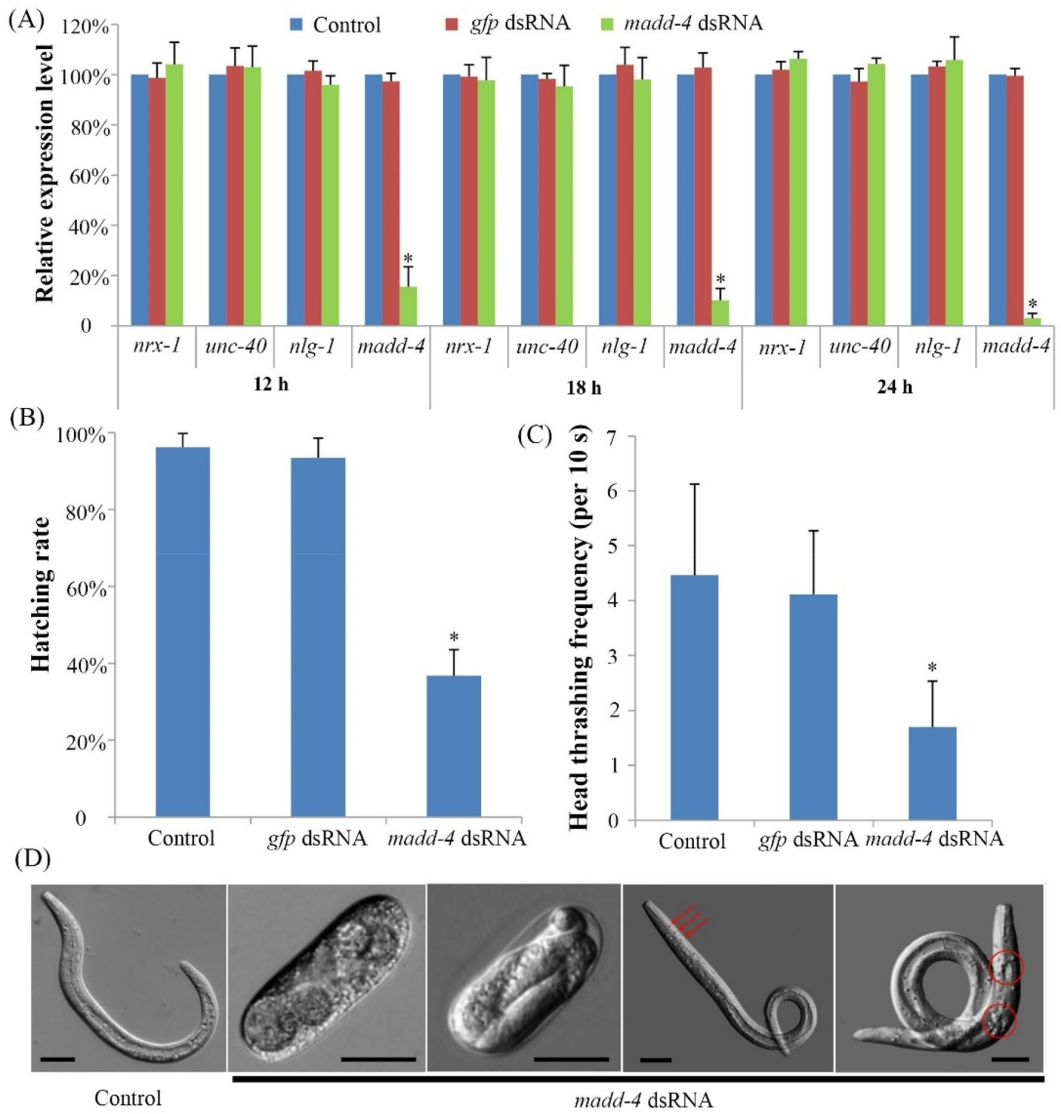
Teratogenic effects of *madd-4* knockdown in *B. xylophilus*. (**A**) mRNA expression levels of *madd-4*, three potential upstream or downstream genes, *nrx-1*, *unc-40*, and *nlg-1* in *B. xylophilus* after RNAi treatment for 12 h, 18 h, and 24 h, respectively. RT-qPCR reactions were performed in triplicate. (**B**) The percentage of hatched embryos in *B. xylophilus* after RNAi treatment for 24 h. (**C**) The number of head thrash per 10 s was recorded to assess the motility of the J2 in *B. xylophilus* after RNAi treatment for 24 h. (**D**) Pictures represented arrested development of embryos and abnormal of J2 in *B. xylophilus* after RNAi treatment for 24 h. Error bars show standard errors. The asterisks show a significant difference (p < 0.05) between the treatments and the controls. Scale bar, 20 μm.

To explore the functions of *Bxy-madd-4* during the development of *B. xylophilus*, an RNAi strategy was used to knockdown its mRNA expression on the synchronized embryos. By comparing the expression levels of *Bxy-madd-4* in the embryos soaked in the dsRNA and the control, showed RNAi led to a reduction of 84.4%, 89.8%, and 97.0% after treatment for 12 h, 18 h, and 24 h, respectively (Fig. 8A). For the three potential upstream or downstream genes, *nrx-1*, *unc-40*, and *nlg-1*, the mRNA expression levels were not significantly reduced after RNAi treatment for 12 h, 18 h, and 24 h (Fig. 8A). RNAi indicated that only 36.8% of the *Bxy-madd-4* dsRNA treated embryos were hatched, whereas almost 100% of the hatching rates were observed in the control groups (Fig. 8B). Moreover, in the *Bxy-madd-4* dsRNA treated group, the unhatched embryos were arrested during the development and lysed, whereas 71.6% of the hatched J2 were abnormal, such as particles on the body surface and concave tissues (Fig. 8D). The number of head thrash per 10 s was recorded to assess the influence of RNAi on the motility in *B. xylophilus*. In the *Bxy-madd-4* dsRNA treatment group, the head thrashing frequency was 1.72 times per 10 s in the hatched J2 (Fig. 8C). In contrast, they were 4.4 times and 4.2 times per 10 s for the hatched J2 in the non-dsRNA treated control and *gfp* dsRNA control, respectively (Fig. 8C).

## Discussion

Previous studies suggested that high light intensity could suppress the development of pine wilt disease^12^. Here, we found the blue light interfering with embryogenesis of the causal agent, *B. xylophilus*, and the transcriptome analysis found that the blue light strongly suppressed the expression level of *madd-4* in the nematode. Furthermore, we validated that the expression level of *Bxy-madd-4* was suppressed by the blue light using RT-qPCR. Meanwhile, knockdown of *Bxy-madd-4* expression found that it played crucial roles in the embryonic development of *B. xylophilus*. These results imply that blue light can suppress the development of pine wilt disease through a negatively regulated expression level of *madd-4* in *B. xylophilus*.

The functional motifs in Bxy-MADD-4, TSP1 motif repeats, and IG-like domains have high homology in *C. elegans*^17^. Many studies have demonstrated the functional complexity of TSP1, such as promoting cell adhesion and proliferation^22^. The IG-like domain is probably the most widespread in animals and involves binding functions^23^. In *C. elegans*, the IG-like domain plays a key role for MADD-4 interaction with NLG-1, which is believed to interact with the presynaptic neurexin protein to mediate heterophilic adhesion^21,24^. These reports imply that Bxy-MADD-4 may play an important role in cell proliferation, tissue expansion, and the synaptic junction of *B. xylophilus*. Bxy-MADD-4 has a signal peptide located at the N-terminus, a critical feature for a secreted protein with distinct physicochemical properties^25^. Phylogenetic analysis indicated that apparent homologues of the isolated ADAMTSL1 from all the nematode species and the Bxy-MADD-4 displays the highest level of sequence homology with the ADAMTSL1 of the dog parasite *T. canis*. ADAMTSL1, also known as punctin, is the first one of ADAMTSL family^26^. ADAMTSL1 is a type of secreted glycoprotein located in the cytoplasmic matrix, but the function remains unclear. In *C. elegans*, *madd-4A* and *madd-4B* had similar expression patterns^17^. However, in *B. xylophilus*, *Bxy-madd-4A* and *Bxy-madd-4B* showed different spatial and temporal expression patterns, *Bxy-madd-4A* was detected nearly throughout the whole body over the course of ontogeny and *Bxy-madd-4B* was detectable only in two tiny dots. Therefore, we speculate that *Bxy-madd-4A* plays a major role in *B. xylophilus*.

For vast majority of living things, the ability to sense different kinds of light sources is essential for survival. There are six photoreceptor families have been widely acknowledged to mediate light signal transduction in nature^15^. However, it is generally believed that nematodes do not become sensitive to the visible light in the past, because they do not possess eyes. In recent years, two light responses, light avoidance and light sensitivity, were found in nematodes^14^. Cyclic adenosine monophosphate and diacylglycerol, the two signals, control the avoidance behaviors of *C. elegans* in ambient light. And, LITE-1, a member of gustatory receptor family, controls avoidance behaviors of the nematode in short-wavelength light, and ultraviolet light^27^. LITE-1 is a distinct type of photoreceptor, unlike the above six photoreceptors using the prosthetic chromophore to trap photons, but strictly depending on the protein conformation to absorb photon^28–30^. LITE-1 can also sense chemical signals, for instance, H_2_O_2_^31^. Here, we discovered that *madd-4*, a member of the ADAMTSL, is regulated by blue light in *B. xylophilus*. MADD-4 is the only one of ADAMTSL family members for which function has been studied. In *C. elegans*, MADD-4 is secreted by the dorsal and ventral nerve cords, and its mutants have remarkable defects in the muscle arm expansion^17^. However, it is not yet known whether the regulation is under the control of photoreceptor or chemoreceptor proteins. A follow-up study of possible mechanism for the light-driven regulation is being planned.

Due to the gene knockout technology that has remained elusive in *B. xylophilus*, a knockdown strategy, RNAi was used to investigate the roles of *Bxy-madd-4* in the current study. Our results are in full agreement with the previous reports that 24 h is sufficient time to achieve a statistically significant reduction of over 80% in gene expression in *B. xylophilus*^32–34^. However, the expression of *Bxy-madd-4* could also be reduced firmly using the soaking time of 12 h and 18 h, suggesting that the duration of soaking could be shortened in some experiments. Through the gene knockdown of *Bxy-madd-4*, the transcription levels of the three potential upstream or downstream genes (*nrx-1*, *unc-40*, and *nlg-1*) did not have any significant differences. These findings suggest that the regulation of *Bxy-madd-4* by blue light in *B. xylophilus* might be based on the control of chemical signals or their relationships is different from those in *C. elegans*. *Bxy-madd-4* knockdown severely inhibited the embryogenesis with approximately 73% of embryos arrested at various development stages, indicating that *Bxy*-*madd-4* is indispensable for embryogenesis in *B. xylophilus*. Knockdown of *Bxy-madd-4* interfered with embryo development of *B. xylophilus* like that of blue light treatment. Moreover, successful knockdown of *Bxy-madd-4* resulted immobility-defective at the J2. We speculate RNAi of *madd-4* might cause defects in muscle arm expansion in *B. xylophilus*, as found in *C. elegans*^17^. Similarly, prolonged exposure to blue light resulted in locomotor impairment in *Drosophila melanogaster*, but not led to degeneration in the retina^35^. Hence, we infer that blue light might suppress the muscle membrane extension through a negatively regulated expression level of *madd-4* in the fly.

In a summary, our data showed that *Bxy*-*madd-4* is a novel negatively light-suppressed gene and plays a key role in embryogenesis. In addition, there is no redundant gene that could compensate for the lose of *Bxy*-*madd-4*. Our results also showed that 18 h, even 12 h is sufficient soaking time to achieve a reduction of over 80% in target gene expression in RNAi experiments, implying that over 24 h duration of soaking is not strictly required in some experiments. However, knocking down of *Bxy-madd-4* expression did not lead to changes in the transcription levels of the upstream or downstream genes (*nrx-1*, *unc-40*, and *nlg-1*), indicating their relationships in *B. xylophilus* may be different from those in *C. elegans*. Due to no effective countermeasures have developed against this destructive invasive nematode, these findings may provide a promising hint for control of the species by blocking ontogenesis.

## Methods

### Biological materials

*B. xylophilus* strain used in the current study was initially isolated from the wilted *Pinus massoniana* in China, and then raised on the mycelia of *Botrytis cinerea* (non-sporulating strain) grown on potato dextrose agar (PDA) Petri dishes at 25 °C. *C. elegans* N2 strain was raised on nematode growth medium Petri dishes inoculated with *Escherichia coli* OP50 strain at 20 °C^36^. Nematodes were extracted from the Petri dishes via Baermann funnel technique^37^. In order to obtain synchronized eggs, the extracted mixed-stage nematodes were switched to a new dish with sterile water and placed in the dark at 25 °C for 0.5 h to lay eggs. The upper layer of water and nematodes were abandoned very carefully, and the synchronized eggs sticked to the bottom of the Petri dishes^7^. The synchronized eggs were incubated at 25 °C for 24 h in the dark in the absence of food to obtain synchronized J2. Then, the synchronized J2 were collected and transferred on the mycelia of *B. cinerea* at 25 °C in the dark in the PDA plates for 48 h to collect synchronized male and female J4 under an optical microscope Zeiss Observer (Carl Zeiss, Oberkochen, Germany). To obtain virgin adult males and females, the synchronized male and female J4 were cultured on the *B. cinerea* at 25 °C for 24 h, respectively^38^.

### *B. xylophilus* under different light sources

To investigate the effects of different light resources on the development of *B. xylophilus*, 10 pairs of virgin adult males and females (10♀ + 10♂) were inoculated onto the mycelia of *B. cinerea* and placed under different light-emitting diode (LED), including red, orange, yellow, green, blue, violet and white (1000 lx), at 25 °C for 7 days. Besides, dark was used as a control. The effects of the different light sources on the development of *B. xylophilus* were assessed by comparative fecundity between the dark and the different light sources treated nematodes.

### Hatching rate under blue light

To assess the effects of blue light on the embryogenesis of *B. xylophilus*, synchronized eggs were placed under blue LED (1000 lx) for 10 different time periods, from 6 to 15 h, and then they were transferred to the dark incubated at 25 °C. Each experiment contains ~ 400 synchronized eggs with nine replicates. Synchronized eggs incubated at dark used as control. The hatching rate of *B. xylophilus* was recorded by the Zeiss Inverted Observer A1 microscope coupled with AxioCamMRc camera (Carl Zeiss, Oberkochen, Germany).

### *Bxy-madd-4* sequence cloning and analysis

The splign program was utilized to demonstrate the gene structure using the full transcript sequence of *Bxy-madd-4* against the genome of *B. xylophilus*. RNA was isolated from the nematodes with TRIzol reagent (Thermo Fisher Scientific Inc., Waltham, USA). cDNA was synthesized by reverse transcription with Prime Script RT reagent Kit (Takara Bio Inc., Shiga, Japan). Partial coding region of *Bxy-madd-4* was amplified from the cDNA by its specific primers (Supplementary Table S2). Products of the amplification were cloned into the pGEM-T vector (Promega, Madison, USA) and sequenced. The presence of conserved structural motifs in Bxy-MADD-4 was predicted using the Scan Prosite tool (https://prosite.expasy.org/). Transmembrane structures and signal peptides of Bxy-MADD-4 were predicted using MHMM and SignalP. Physical and chemical properties of Bxy-MADD-4 were determined using ProtParam.

### RT-qPCR and RNA-seq to detect the expression level of *Bxy-madd-4* under blue-light

RT-qPCR and RNA-seq were utilized to verify the expression level of *Bxy-madd-4* under blue LED during embryogenesis. In brief, synchronized eggs were placed under blue LED (1000 lx) at three different time periods, 7, 10, and 13 h, at 25 °C. Then the blue light treated eggs were transferred to the dark incubated at 25 °C. Meanwhile, synchronized eggs incubated at dark were used as controls. Each experiment was conducted with three replicates. RNA was extracted and reversed as described above. RT-qPCR was conducted with the SYBR Premix Kit (Takara Bio Inc., Shiga, Japan) using the Mx3000P qPCR System (Agilent, USA). All the RT-qPCR reactions were conducted with three replicates. The *β-actin* (EU100952) and *tbb-2* (MT769316)were used as the endogenous reference genes^39^. Gene-specific primers for reference and target genes were designed by Primer Premier 5 software (Supplementary Table S2). The relative gene expression data were analyzed via the 2^−ΔΔCT^ approach^40^. Besides, FPKM of *Bxy-madd-4* was extracted from our recently sequenced transcriptome data^16^.

### Construction of transgenic *C. elegans*

To mimic the *Bxy-madd-4* expression patterns in *B. xylophilus*, *Bxy-madd-4A* promoter (*Bxy-madd-4Ap*) and *Bxy-madd-4B* promoter (*Bxy-madd-4Bp*) were cloned into the expression vector pCFJ90, and injected into *C. elegans*, respectively. The amplification reaction for the promoters were conducted in 50 μl reaction volumes, containing 5 μl of 10 × LA PCR buffer (TAKAR., Dalian, China), 8 μl of 2.5 mmol/l of dNTPs, 5 μl of 25 mmol/l of MgCl_2_, 2.5 U of LA Taq DNA polymerase (TAKAR., Dalian, China), 1 μl of 10 pmol/l of each primer, 1 μl of 20 ng/ml template DNA, and double distilled water to make up the volume to 50 μl. The amplification conditions were as follows: one cycle at 94 °C for 1 min; 40 cycles at 98 °C for 10 s, 60 °C for 40 s, 68 °C for 5 min; and one cycle at 72 °C for 10 min. The amplified products of the promoters were cloned into the pGEM-T Easy vector (Promega, Madison, USA) and verified by sequencing. The expression patterns of Cherry (red) under control of *Bxy-madd-4Ap* and *Bxy-madd-4Bp* during *C. elegans* development. Meanwhile, the reporter gene fluorescent protein (*gfp*; green) was driven by *C. elegans* promoter *myo-2* as a marker for pharynx development. Basing on the *B. xylophilus* genome and full transcript sequences, around 3000 bp genomic DNA sequence upstream of the start codon (ATG) of each isoform was predicted to be the promoter region. Gene-specific primers for the two promoter regions were designed with 15 base extensions homologous to pCFJ90-*Pmyo-2*::Cherry::unc-54utr end (Supplementary Table S2). The genomic DNA of *B. xylophilus* was extracted with QIAamp DNA Mini Kit (QIAGEN, Hilden, Germany) according to the manufacturer's instructions, then *Bxy-madd-4Ap* and *Bxy-madd-4Bp* were amplified by PCR. pCFJ90-*Bxy-madd-4Ap*::mCherry::unc-54utr and pCFJ90-*Bxy-madd-4Bp*::mCherry::unc-54utrwere constructed, purified and injected at 80 ng/μl into the gonad of *C. elegans* hermaphrodites as described in the previous reports, respectively^41,42^. pCFJ90-*Pmyo-2*::GFP::unc-54utr was also injected at 5 ng/μl as a coinjection marker to indicate the location of pharynx development in *C. elegans*^43,44^. The expression profiles of pCFJ90-*Bxy-madd-4Ap*::mCherry::unc-54utr and pCFJ90-*Bxy-madd-4Bp*::mCherry::unc-54utr were observed in the successful transgenic F2 offsprings during different developmental stages (early embryo, late embryo, L1, L2, L3, L4, and adult) with a Leica DM 2500 florescent microscope (Leica, Wetzlar, Germany).

### RNAi of *Bxy-madd-4*

RNAi approach was used to investigate the functions of *Bxy-madd-4* in the embryonic development of *B. xylophilus*. The partial shared (*Bxy-madd-4A* and *Bxy-madd-4B*) coding region of *Bxy-madd-4* (1062 bp) was utilized for dsRNA synthesis with MEGAscript Transcription Kit (Ambion Inc., Austin, USA). *gfp* was used as an exogenous control^45^. The quality and quantity of the synthesized dsRNA were determined by NanoDrop (Thermo Fisher Scientific Inc., Waltham, USA). Synchronized newly laid eggs of *B. xylophilus* were soaked in the 0.8 µg/ml of dsRNA of *Bxy-madd-4* and *gfp* at 25 °C for 12 h, 18 h, and 24 h in the dark, respectively. In addition, soaking buffer (0.05% gelatin, 5.5 mM KH_2_PO_4_, 2.1 mM NaCl, 4.7 mM NH_4_Cl, 3 mM spermidine) was used as a non-dsRNA control. RNAi-soaking approach was performed based on our previous report^33^. Each experiment was conducted with five replicates. The RNAi efficacy was evaluated by RT-qPCR using the 2^−ΔΔCT^ method, as described above. *madd-4* and neurexin (*nrx-1*) play partially redundant role to recruit neuroligin (*nlg-1*) to synapses^21^. The activity of MADD-4 is dependent on the netrin receptor, UNC-40^17^. Therefore, the expression profiles of the upstream and downstream genes (*nrx-1*, GenBank accession number MW349429; *nlg-1*, GenBank accession number MW349430; *unc-40*, Gen Bank accession number MW349431) were also investigated. The specific primers of the above genes were designed by Primer Premier 5 software (Supplementary Table S2). Functions of *Bxy-madd-4* were assessed by comparative analysis on the development statuses between the controls and the RNAi-treated nematodes. The unhatched eggs and J2 were scored under an optical microscope Zeiss Observer. The number of head thrash per 10 s was recorded to assess the motility. The motion of *B. xylophilus* was recorded by the Zeiss Inverted Observer A1microscope coupled with AxioCamMRc camera (Carl Zeiss, Oberkochen, Germany).

### Statistics

The number of offsprings, relative expression levels, hatching rates, head thrashing frequencies were statistically analyzed and performed by SPSS 22.0 based on the one-way ANOVA, respectively (SPSS Inc., Chicago, IL, United States). Data were considered statistically significant when p < 0.05. Each sample has at least three biological replicates. All RT-qPCRs were carried out in triplicate.

## Supplementary Information

Supplementary Information.

## Data Availability

All the datasets generated in the current study are available from the corresponding author upon request.
