# Supplementary material for: madd-4 plays a critical role in light against Bursaphelenchus xylophilus
Source: Sci Rep. 2022 Aug 30;12:14796. doi: 10.1038/s41598-022-19263-9 (PMC9427778; doi:10.1038/s41598-022-19263-9)
Supplement: Supplementary file 1 — Supplementary Information. [file 41598_2022_19263_MOESM1_ESM.pdf]

Table S1 The expression patterns of *Bxy-madd-4* archiving from RNA-seq data. Each sample has three biological replicates. FPKM, fragments per kilobase of exon model per million reads mapped.

Samples		Expression levels of *Bxy-madd-4* (FPKM)
Dark	7h	39.7446
		39.2164
		32.4437
	10h	244.391
		237.177
		238.952
	13h	186.024
		164.466
		165.452
Blue light	7h	29.4735
		23.6648
		22.7991
	10h	36.8083
		35.5451
		25.6749
	13h	38.2549
		94.6113
		66.248

Table S2 Primers used in this study. F, forward; R, reverse.

**Primer**	**Sequence (5′-3′)**
Cloning *madd-4*	*madd-4* F: ccgagtggtccgaatgct<br>*madd-4* R: ctccgtttccagacactttcat
RT-qPCR	*madd-4* F: cccgatgcccaatgtccactacc<br>*madd-4* R: accactctatgctgttcaccttgcc<br>*nlg-1* F: gttgagtaagatccttccttcgttcg<br>*nlg-1* R: gagtctattatttggctgtgtgatggg<br>*unc-40* F: gtaagccgaaagggatcttctcaatgg<br>*unc-40* R: gggcagtgggtgagtttggaacgacag<br>*nrx-1* F: gcacaccttacgacttctctg<br>*nrx-1* R: agatccttgctgctgacattg<br>*tbb-2* F: gaatccaacatgaacgatct<br>*tbb-2* R: cttcgtattcaccatcatct<br>*β-actin* F: cgcaaatactccgtctggattgg<br>*β-actin* R: ttcgtcgtactcttgcttggaga
Cloning promoter	*madd-4A* F: tgataccagcggataacaattttctaatcgacgtcgacgtatg<br>*madd-4A* R: tttgtacaaacttgtcattttcattcattttacactttgtttcattgttttttttcc<br>*madd-4B* F: tgataccagcggataagctccattaagaagtggagaattggt<br>*madd-4* B R: tttgtacaaacttgtcattgtgctcaagttccaaaattccaaaac
Synthesis dsRAN	*madd-4* F: taatacgactcactatagggagaccgagtggtccgaatgct<br>*madd-4* R: taatacgactcactatagggagactccgtttccagacacttt

	gfp F: taatacgactcactatagggagaaaaggagaagaacttttca
	gfp R: taatacgactcactatagggagactgttacaaactcaagaag
